# Characteristics of Body Composition Estimated by Air-Displacement Plethysmography in Chinese Preschool Children

**DOI:** 10.3389/fpubh.2022.926819

**Published:** 2022-06-03

**Authors:** Fangfang Chen, Jing Wang, Junting Liu, Guimin Huang, Dongqing Hou, Zijun Liao, Ting Zhang, Gongshu Liu, Xianghui Xie, Jun Tai

**Affiliations:** ^1^Department of Epidemiology, Capital Institute of Pediatrics, Beijing, China; ^2^Research Project Group, Tianjin Women's and Children's Health Center, Tianjin, China

**Keywords:** body composition, preschool children, air displacement plethysmography, fat mass, Chinese

## Abstract

**Objective:**

To describe the characteristics of body composition by air-displacement plethysmography (ADP) among Chinese preschool children.

**Methods:**

Preschool children were recruited from three kindergartens. Adiposity indices were evaluated using the ADP method. BMI, fat mass index (FMI), fat-free mass index (FFMI) and waist-to-height ratio (WHtR) were calculated. Overweight and obesity were diagnosed using the WHO reference. Analyses were executed by SPSS and MedCalc software. Smoothed curves were constructed using the lambda-mu-sigma (LMS) method.

**Results:**

This study evaluated the growth trend for body composition of ADP-based body fat indices based on a relatively large sample of preschool children, the first ever reported in China. A total of 1,011 children aged 3–5 years comprised our study population. BMI and FFMI increased with age, but the slope (*P* = 0.710) and y intercept (*P* = 0.132) in the BMI trend analysis demonstrated no differences between boys and girls. For the FFMI trend lines, the slope was significantly higher for boys than for girls (*P* = 0.013). The percentage of fat mass (FM%), FMI, and WHtR were negatively correlated with age for both sexes, except for FMI in girls (*P* = 0.094). The 95% CI regression lines for FM% according to different weight statuses intersected.

**Conclusions:**

ADP is applicable to estimating body composition among Chinese preschool children. Misclassifications might occur when overweight/obese status is defined based on surrogate indices.

## Introduction

Overweight and obesity impose heavy clinical and public health burdens worldwide (1). Trend in body mass index (BMI) from 1975 to 2016 showed that while the rising trends in children's BMIs have plateaued at relatively high levels in many high-income countries, they have accelerated in parts of Asia (2). The prevalence of obesity among Chinese preschool children increased continuously from 1986 to 2016, especially after 3 years of age (3).

Obesity is not excess weight, while it is excess adipose tissue (4). Asians tend to have a higher percent body fat at a lower BMI than Westerns, and the fat store patterns of ethnic specificity are partly determined by genetics (5). Anthropometric measurement indices such as BMI and waist circumference, are widely used to evaluate adiposity owing to their feasibility and low cost, but their use is limited because they do not provide indications of overall body composition (6). A higher BMI may arise not only from greater body fat but also from higher lean mass and/or bone mass, which making BMI an imperfect measure of adiposity (7, 8).

Air-displacement plethysmography (ADP) has been used to measure human body composition for nearly a century, but was not developed into a viable system for routine use until the mid-1990s (9, 10). The commercially available system for ADP named BOD POD (Body Composition System; COSMED USA, Concord, CA, United States) is a new practical alternative to those more traditional body-composition methods, such as dual-energy X-ray absorptiometry (DXA), hydrostatic weighing and measurement of total body water by isotope dilution (11), and it offers several advantages, including a quick, comfortable, automated, non-invasive, and safe measurement process (12). However, as with any novel technology, it is important to establish its validity and practicality in various populations.

Asians are known to possess a different amount and distribution of body fat than Caucasians of similar BMI (13, 14). Many studies have focused on youth and performed direct measurements of body composition, including skin fold thickness, bioelectrical impedance analysis and the DXA method (15–17). Our previous studies showed that increases in BMI during preschool childhood are mainly due to greater fat-free mass (predominantly muscle) rather than fat mass based on DXA-measured body composition (17). ADP is a reliable method for determining FM% in infants, children and adults (18). Few studies have evaluated body composition using the ADP method and estimate the validation among children.

Ma et al. (19) reported the results of body composition using the ADP system (PEAPOD) in Chinese infants (0.4–21.7 weeks). Deurenberg et al. (20) performed the research among 7- to 12-year-old children in Beijing with body fat measured using densitometry (underwater weighing). While no previous work seems to have examined body composition by the ADP method in Chinese preschoolers, and no investigators have yet assessed the validity of BODPOD measurements in Chinese children. To fill this gap, we designed the present study to assess body composition by ADP measurements in Chinese preschool children. Furthermore, based on the estimation of body composition, we also aimed to evaluate whether misclassification occurs when weight status is defined according to surrogate indices.

## Materials and Methods

This study was carried out by the Tianjin Women's and Children's Health Center and Capital Institute of Pediatrics (CIP) in Tianjin, which is a province-level municipality in northern China. The study was approved by the Institutional Review Board of the Tianjin Women's and Children's Health Center (BGI-IRB 17116-201711), and written informed consent was obtained from the parents of each subject.

### Participants

Preschool children were recruited from three kindergartens in the Jinghai district in Tianjin between September 2020 and December 2020, and parental consent was obtained upon recruitment. The exclusion criteria were (1) the inability to provide informed parental consent; (2) any condition or chronic disease or use of any drug known to affect growth and development; and (3) acute diseases that prohibited children from participating in physical examinations.

### Anthropometric and Body-Composition Measurements of Children

Body weight, fat mass, fat-free mass, percentage of fat mass (FM%), and body volume and density were evaluated by a commercially available system for ADP known by the trade name BOD POD. We used the pediatric option (including a custom seat that was secured to the testing chamber/seat of the BOD POD) and a prototype software version. In this procedure, body mass was measured using an electronic scale, and body volume was assessed in a closed chamber utilizing the relationship between pressure and volume. The principle of measurement that we applied to children was the same as that for adults (21). The subject entered the BOD POD system without shoes in a tightfitting swimsuit and a swimming cap, and their total body volume was measured. Volume measurements were always performed in triplicate and strictly according to the manufacturer's instructions. Body volume was corrected for surface area artifacts and thoracic gas volume. Surface area artifacts and thoracic gas volume were estimated based on the equations that were developed and built in the machine. Calculations of body composition were carried out as described by Fields and Allison (22).

For each child recruited from kindergarten, height and waist circumference were measured according to the standardized protocol. The height of the children was measured to the nearest 10th of a centimeter without shoes by trained staff with wall-mounted stadiometers. Waist circumference was measured when the child stood comfortably with his or her weight evenly distributed on both feet. The measurement was taken along the midaxillary line at the midpoint between the inferior margin of the last rib and the crest of the ilium and in a horizontal plane. Each landmark was palpated and marked, and the midpoint was determined with an inelastic measuring tape and marked. The observer sat by the side of the child and fit the tape snugly (but not so tightly as to compress underlying soft tissues), and waist circumference was measured at the end of normal expiration to the nearest 10th of a centimeter.

We calculated BMI for each subject as body weight in kilograms divided by height in meters squared. Fat mass index (FMI) and fat-free mass index (FFMI) were calculated as fat mass in kilograms and fat-free mass in kilograms, respectively, divided by height in meters squared. The waist-to-height ratio (WHtR) was calculated as the ratio of waist circumference to height.

### Classifications of Overweight and Obese

For children under 5 years of age (60 months), children above +2 standard deviations (SDs) were described as overweight, and those above +3 SDs were depicted as obese based on BMI-for-age and weight-for-height according to the WHO growth standards for children aged 0–5 years (23, 24). For children above 60 months of age, overweight and obese were defined by the WHO reference for children aged 5–19 years (25) based on BMI-for-age (+1 SD and +2 SD).

### Statistical Analyses

Continuous variables are presented as the means ± *SD*. Comparisons of characteristics were conducted using one-way ANOVA or chi-squared tests between boys and girls, and the correlations among BMI, FM%, FMI, FFMI, and WHtR for age were carried out with linear regression. Slopes and intercepts were compared by sex to assess significant differences. A two-tailed *P*-Value was considered significant at <0.05. We performed statistical calculations using SPSS 20.0 (SPSS Inc., Chicago, IL, United States) and MedCalc version 20.0 (MedCalc Software Ltd., Ostend, Belgium).

Smoothed curves for adiposity indices comprising BMI, FM%, FMI, FFMI, and WHtR were constructed for boys and girls separately using the lambda-mu-sigma (LMS) method (LMS Chart Maker Pro Version 2.54, Medical Research Council, London, United Kingdom). This method converted skewed distributions to normal using the maximum likelihood method; adjusted the curves for medians (*M*), the coefficient of variation (*S*), and Box–Cox power (*L*); and smoothed the percentile curves of adiposity indices by cubic natural smoothing spline functions (26, 27). Percentile curves of adiposity indices were plotted with Origin 9 (OriginLab Corporation, Northampton, MA, United States). *Z* values were calculated with the following equation: *Z* = [(*X*/*M*) ^*L*^ – 1]/LS, (*L* ≠ 0), where *X* is the adiposity index, *L* is the power transformation, *M* is the median value, and *S* is the population SD.

## Results

### Participants and Characteristics

Table 1 presents subject characteristics separated by sex. A total of 1,011 children (526 boys and 485 girls) aged 3–5 years comprised the study population. We noted no significant differences in age, weight, height, BMI, waist circumference, WHtR, or FM% between the sexes, nor were there differences in the prevalence rates of overweight or obesity between boys and girls.

**Table 1:** Characteristics of study population.

	**Total** **(*n* = 1,011)**	**Boys** **(*n* = 526)**	**Girls** **(*n* = 485)**	*P* ^*^
Age (years, mean ± SD)	4.5 ± 0.8	4.5 ± 0.8	4.5 ± 0.8	0.864
Weight (kg, mean ± SD)	18.4 ± 4.1	18.7 ± 4.2	18.0 ± 3.9	0.163
Height (cm, mean ± SD)	107.9 ± 7.3	108.5 ± 7.5	107.2 ± 7.0	0.128
BMI (kg/m^2^, mean ± SD)	15.7 ± 1.9	15.7 ± 1.9	15.6 ± 1.9	0.717
Waist circumference (cm, mean ± SD)	53.0 ± 5.3	53.3 ± 5.5	52.7 ± 5.2	0.313
WHtR (mean ± SD)	0.49 ± 0.04	0.49 ± 0.04	0.49 ± 0.04	0.803
FM% (%, mean ± SD)	21.3 ± 7.2	21.5 ± 7.2	21.1 ± 7.3	0.786
FMI (kg/m^2^, mean ± SD)	3.4 ± 1.5	3.5 ± 1.5	3.4 ± 1.5	0.768
FFMI (kg/m^2^, mean ± SD)	12.3 ± 1.3	12.3 ± 1.4	12.2 ± 1.3	0.230
Overweight^#^ (*n*, %)	91, 9.0%	47, 8.9%	44, 9.1%	0.939
Obese (*n*, %)	60, 5.9%	37, 7.0%	23, 4.7%	0.123

* *P-Values were from tests compared between boys and girls by t-tests in continuous variables and chi-square tests in categorical variables*.

# *Obese not included. Overweight and obese were defined according to the WHO growth standards*.

*BMI, Body mass index; WHtR, Weight height ratio; FM%, Percentage of fat mass; FMI, Fat mass index; FFMI, Fat-free mass index*.

Table 2 presents the characteristics of growth and adiposity indices by age and sex. The x^2^-test for trend showed that the prevalence rates for overweight and obesity increased with age in both boys and girls (all *P*_fortrend_ < 0.001).

**Table 2:** Characteristics of growth and adiposity indices by age and gender.

**Age**	***N***	**Height**	**Weight**	**BMI**	**Waist circumference**	**WHtR**	**FM%**	**FMI**	**FFMI**	**Overweight^#^**	**Obese**
**(years)**		**(cm)**	**(kg)**	**(kg/m^2^)**	**(cm)**		**(%)**	**(kg/m^2^)**	**(kg/m^2^)**	**%**	**%**
**Boys**										
3.0	172	101.5 ± 4.2	15.9 ± 2.1	15.4 ± 1.3	50.3 ± 3.5	0.50 ± 0.03	22.2 ± 7.0	3.5 ± 1.3	11.9 ± 1.2	2.9	0.6
4.0	202	108.3 ± 4.7	18.7 ± 3.4	15.9 ± 2.0	53.6 ± 5.3	0.50 ± 0.04	24.1 ± 6.2	3.9 ± 1.4	12.0 ± 1.3	7.4	5.0
5.0	152	116.7 ± 4.8	21.9 ± 4.7	16.0 ± 2.3	56.1 ± 6.0	0.48 ± 0.04	17.4 ± 6.9	2.9 ± 1.6	13.1 ± 1.3	17.8	17.1
**Girls**										
3.0	166	101.2 ± 3.9	15.7 ± 2.1	15.3 ± 1.4	50.4 ± 3.6	0.50 ± 0.03	20.6 ± 7.0	3.2 ± 1.3	12.1 ± 1.1	2.4	0
4.0	180	106.6 ± 4.2	17.7 ± 2.9	15.5 ± 1.9	52.6 ± 4.5	0.49 ± 0.04	24.0 ± 6.4	3.8 ± 1.4	11.7 ± 1.2	6.7	1.7
5.0	139	115.0 ± 4.9	21.1 ± 4.5	15.9 ± 2.3	55.4 ± 6.1	0.48 ± 0.04	18.1 ± 7.3	3.0 ± 1.6	12.9 ± 1.3	20.1	14.4

# *Obese not included. Overweight and obese were defined according to the WHO growth standards*.

*BMI, Body mass index; WHtR, Weight height ratio; FM%, Percentage of fat mass; FMI, Fat mass index; FFMI, Fat-free mass index*.

### Body Composition Among Preschool Children

Trends of body composition indices changed with age, as determined by linear regression analysis and are presented in Table 3. Although BMI and FFMI were significantly positively correlated with age for both sexes, the slope (*P* = 0.710) and y intercept (*P* = 0.132) for the BMI trend demonstrated no differences between the sexes. Regarding the FFMI trend lines, although the y intercept showed no difference between boys and girls (*P* = 0.337), the slope was significantly higher in boys than in girls (*P* = 0.013). FM%, FMI, and WHtR were significantly negatively correlated with age for both sexes, except for FMI in girls (*P* = 0.094). FM% declined with age faster in boys than in girls (the difference in slope was −1.191, *P* = 0.025). The slope and y intercept analysis of the FMI (slope, *P* = 0.108; y intercept, *P* = 0.258) and WHtR (slope, *P* = 0.758; y intercept, *P* = 0.924) trends exhibited no differences between boys and girls.

**Table 3:** Trend of body composition indices changed with age by linear regression analysis.

	**β**	**95%CI**		***P***
**Boys (*****n*** **=** **526)**				
BMI	0.325	0.130	0.521	0.001
WHtR	−0.007	−0.011	−0.004	<0.001
FM%	−2.586	−3.288	−1.884	<0.001
FMI	−0.309	−0.457	−0.161	<0.001
FFMI	0.632	0.504	0.760	<0.001
**Girls (*****n*** **=** **485)**				
BMI	0.272	0.070	0.475	0.009
WHtR	−0.008	−0.012	−0.004	<0.001
FM%	−1.395	−2.166	−0.624	<0.001
FMI	−0.133	−0.289	0.023	0.094
FFMI	0.399	0.267	0.531	<0.001

*Body composition indices as dependent variable, exact age as independent variable*.

*BMI, Body mass index; WHtR, Weight height ratio; FM%, Percentage of fat mass; FMI, Fat mass index; FFMI, Fat-free mass index*.

**Figure 1:**
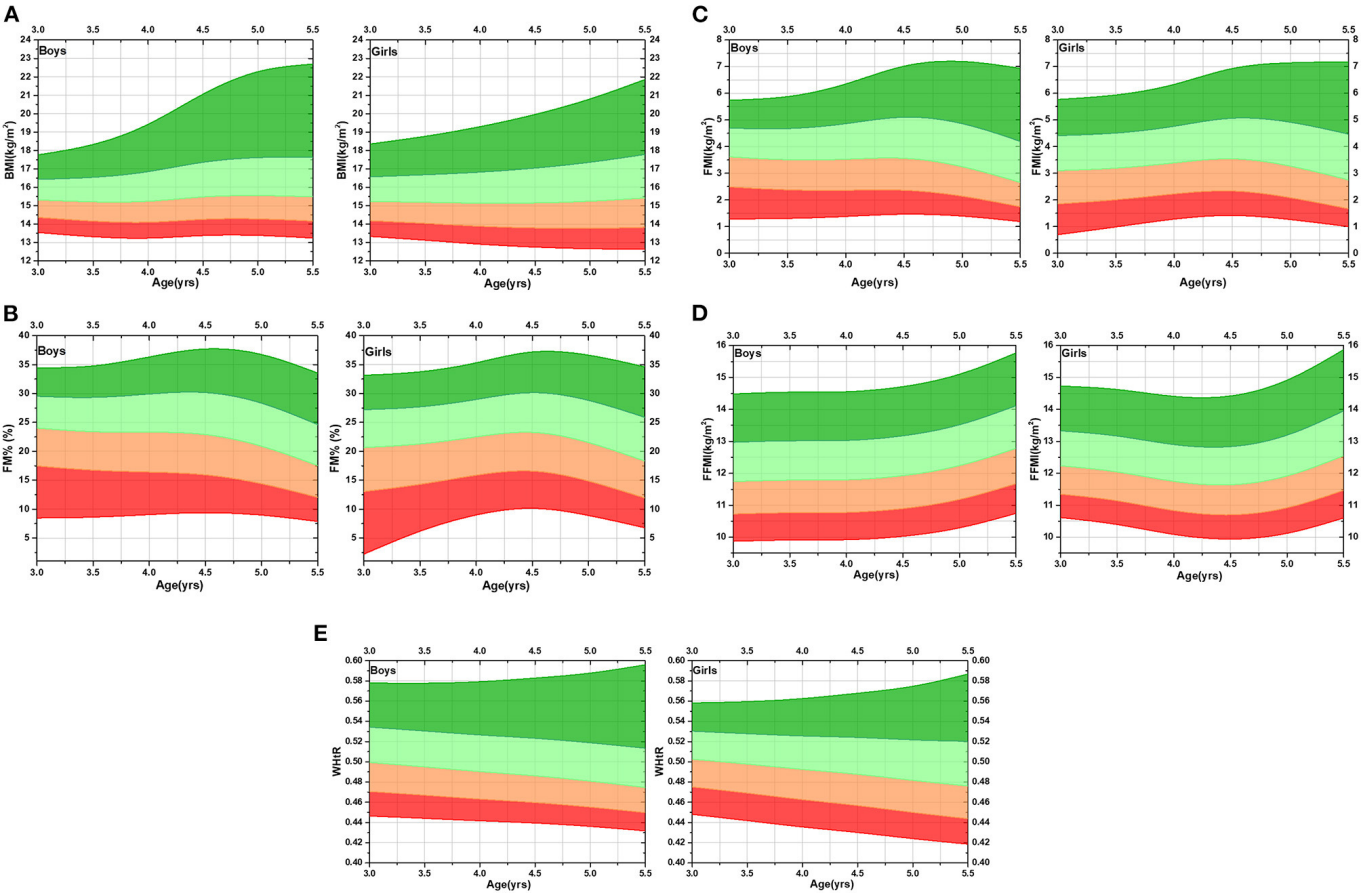
[**(A)** BMI; **(B)** FM%; **(C)** FMI; **(D)** FFMI; **(E)**.WHtR] Reference curves for BMI, FM%, FMI, FFMI, and WHtR of preschool children. Red area, mean – 2 SD of the mean – SD; yellow area, mean – SD of the mean; cyan area, mean to mean + SD; green area, mean + SD to mean + 2 SD. BMI, Body mass index; WHtR, Weight-to-height ratio; FM%, Percentage of fat mass; FMI, Fat mass index; FFMI, Fat-free mass index; SD, Standard deviation.

Figure 1 shows the smoothed LMS curves for the mean, mean ± 1 SD, and mean ± 2 SD for all adiposity indices in both boys and girls. Although the mean BMI value increased with age gradually between 3 and 5 years of age, we noted a marked difference between the FMI and FFMI curves. Overall, FM% and FMI showed a declining trend, especially after 4.5 years of age, while FFMI increased after 4.5 years of age.

### FM% in Different Weight Statuses

**Figure 2:**
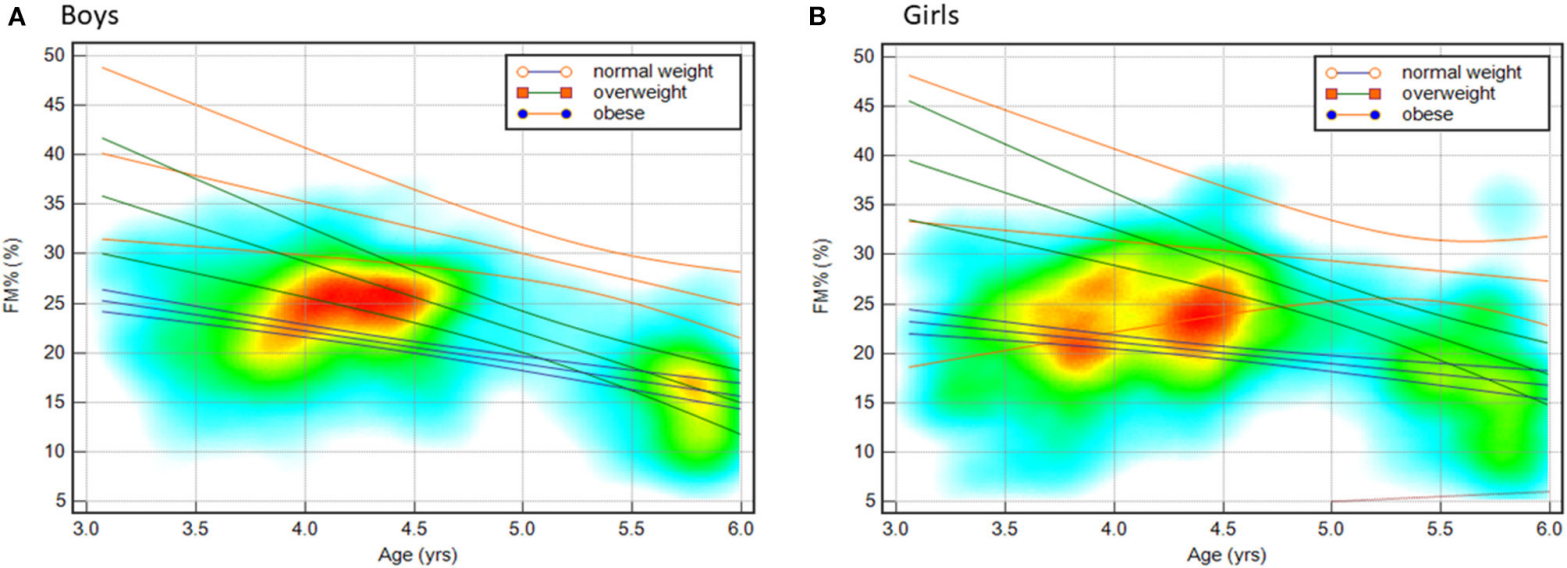
[**(A)** Boys; **(B)** Girls] Scatter-diagram heatmap and regression lines for the percentage of fat mass (FM%) plotted by age for boys **(A)** and girls **(B)**. The blue lines indicate the best-fit line and the upper and lower 95% confidence limits of normal-weight children. The green lines indicate those of overweight children, and the orange lines indicate those of children with obesity.

The percentage of fat mass plotted by age for boys and girls is illustrated in Figure 2, and the regression lines were constructed according to different weight statuses. As shown in the figure, the 95% CI for the regression line of FM% in overweight boys overlapped with that of obese boys 4.5 years of age. The regression lines for obese girls under 5 years of age were dispersed and crossed those of overweight and normal-weight girls. For boys over 4.5 years, the 95% CI regression lines for FM% in obese boys were all higher than those for overweight and normal-weight boys. For girls over 5.5 years of age, the 95% CI regression line for FM% in obese girls was higher than that for overweight and normal-weight girls. In boys over 5 years of age and girls over 5.5 years of age, the 95% CI regression line for FM% for normal weight crossed that of overweight children.

## Discussion

The present study was designed to test the ADP body-composition system in Chinese preschool children and provide a reference for the adiposity indices development of 3–5 years old children, including curves for the mean, mean ± 1 SD, and mean ± 2 SD for BMI, FM%, FMI, FFMI, and WHtR. We found that the mean BMI value increased with age gradually from 3 to 5 years, but fat mass and fat-free mass showed different trends and were even different between the sexes. In boys, the WHtR, FMI, and FM% showed a declining trend, while FFMI increased with age. In girls, the WHtR and FM% also showed declining trends; FFMI increased with age, while FMI did not change with age significantly.

Body mass index has been widely used as a simple surrogate index of defining overweight and obesity and has been shown to be associated with adverse metabolic outcomes. However, it cannot differentiate between fat mass and fat-free mass (28). Data from 387 children participated in the Fels Longitudinal Study had their body composition (evaluated by hydrodensitometry) followed over 10 years, which reported that observed increases in BMI during childhood are mainly due to greater fat-free mass rather than fat mass (29). Oliveros et al. (30) created the concept of “normal weight obesity,” where individuals are classified as being normal weight based on traditional BMI classifications while at the same time having a high body fat percentage. Our previous study explored the impact of body composition on abnormal metabolic phenotypes (31) and found that the amount and location of body fat should still be considered when assessing the risk of metabolic disorder even in children with normal weight. A systematic review and meta-analysis found that BMI exhibited high specificity but low sensitivity in detecting excess adiposity and failed to identify over a quarter of children with an excess body-fat percentage (8). Our classification of weight status as normal weight, overweight, and obese was based on BMI-for-age and weight-for-height according to the WHO growth standards, which were all based on surrogate indicators that possess limitations in distinguishing fat mass from fat-free mass. Figure 2 shows the limitations from the perspective of distinguishing FM% for preschool children. To distinguish FM%, the classifications of overweight and obese were good indicators for boys over 4.5 years of age and girls over 5.5 years of age, but misclassification may occur among younger preschool children. Simultaneously, because of the overlap between the sexes with respect to the 95% CI regression line for FM%, misclassification may also occur when children are diagnosed as normal weight and overweight in boys over 5 years of age and girls over 5.5 years of age.

Due to the limitations of BMI in detecting obesity in children, circumferences can be applied as complementary measures of adiposity, with waist circumference used as an indication of central obesity to identify children at risk of obesity-related metabolic disorders morbidity later in life (32). Waist circumference is also age- and sex-dependent in children, and has been found to be nearly as effective in determining excess adiposity as BMI. A practically solution to the question of age, sex, and height influences on waist circumference is the use of an ideal WHtR (33). In recent years, the WHtR has garnered interest, and it has been found to accurately predict central adiposity (34, 35); it is also considered a better predictor of adiposity than either waist circumference or BMI (35). In this study, waist circumference and WHtR showed no differences between boys and girls aged 3–5 years, and although waist circumference and height both increased with age, WHtR diminished with age in preschool children—particularly for girls. The results of our study also showed that, based on simple anthropometry, BMI increased with age, while WHtR decreased with age in children aged 3–5 years. However, changes in BMI include changes in both FMI and FFMI. FFMI increased with age in both boys and girls, and FMI decreased with age in boys, which was not completely consistent with the trend with age in BMI. This study indicated that when conditions permit, it is better to accurately evaluate the body composition of preschool children than only based on surrogate indices when evaluating obesity.

BOD POD is a reliable and valid technique that can quickly and safely assess body composition in a wide range of populations (11). As a novel method, it was demonstrated that BOD POD with pediatric attachment was accurate, reliable, precise, and unbiased in estimating percentage body fat (12), and the applicability of using in children aged 2–6 years has been validated (22). Our results, however, were not completely congruent with those of other studies, especially with those using different methods to assess children's body fat. The mean value for FM% was 21.3% for 3–5-year-olds in our study, less than that shown in the United States (25.6%) among 2–6-year-olds as estimated by BOD POD (22). A study of body composition assessed by the ADP method in preschool children from Singapore's multiethnic Asian population (36) showed that the mean FMI was 3.7 ± 1.3 kg/m^2^, which is similar to our study (mean FMI, 3.4 ± 1.5 kg/m^2^). The FM% results were obviously lower than those in another study carried out in China, in which body composition was measured by DXA (FM% of children aged 3.5–5.5 years, 34.3%−35.4%) (37), and they were similar to the results from the United States using the DXA method (FM% of children aged 3–5 years, 17.6%−25.4%) (38). We conjecture that age range, population source, sex-composition ratio, and differing body-composition estimation methods might have contributed to the study differences.

The major strength of this study was our evaluation of the growth trend for body composition of ADP-based body fat indices based on a relatively large sample of 3–5 years old children, which seemed the first ever reported in China. Another key strength of these newly created charts was the provision of LMS values, an ADP-based estimation of body fat that could be easily converted to a Z score.

This study possessed several limitations. ADP with the pediatric option was accurate and without bias in estimating % fat in children 2–6 years of age; however, it was unable to distinguish between different body fat locations in the body (39). We therefore could not describe growth trends with respect to the composition of different body areas among preschool children. Another potential limitation of this study was the potential limited relevance of our urban data to that of rural Tianjin children and adolescents, which remains to be investigated in future research. Finally, current reference charts are based on cross-sectional data, and a more appropriate assessment of an individual's body composition growth would be based on charts generated from a longitudinal study sample.

## Conclusion

We evaluated the growth trend for body composition in a set of ADP-based body fat indices—including BMI, FM%, FMI, FFMI, and WHtR—among Chinese children 3–5 years of age. The ADP with the pediatric option is thus applicable to estimating body composition among Chinese preschool children. We also demonstrated that by ignoring body-composition evaluations of preschool children, misclassification will occur when weight status is defined according to surrogate indices.

## Data Availability Statement

The original contributions presented in the study are included in the article/supplementary material, further inquiries can be directed to the corresponding author/s.

## Ethics Statement

The studies involving human participants were reviewed and approved by the IRB of Tianjin Women's and Children's Health Center (BGI-IRB 17116-201711). Written informed consent to participate in this study was provided by the participants' legal guardian/next of kin.

## Author Contributions

FC conceptualized and designed the study, carried out the analyses, drafted the manuscript, and reviewed and revised the manuscript. XX and JT conceptualized the study, supervised data analyses, and reviewed the manuscript. TZ and GL conceptualized and designed the study and supervised data analyses. JW, JL, GH, DH, and ZL involved in data acquisition and critically reviewed and revised the manuscript. All authors critically reviewed the manuscript for interpretation, intellectual content, and approved the final manuscript as submitted.

## Funding

This work was supported by the National Key Research and Development Program of China (2016YFC1300100), CAMS Initiative for Innovative Medicine (2016-I2M-1-008), Public Service Development and Reform Pilot Project of Beijing Medical Research Institute (BMR2019-11), The Special Fund of the Pediatric Medical Coordinated Development Center of Beijing Hospitals Authority (XTZD20180402), and Beijing Municipal Administration of Hospitals Incubating Program (Px2022052).

## Conflict of Interest

The authors declare that the research was conducted in the absence of any commercial or financial relationships that could be construed as a potential conflict of interest.

## Publisher's Note

All claims expressed in this article are solely those of the authors and do not necessarily represent those of their affiliated organizations, or those of the publisher, the editors and the reviewers. Any product that may be evaluated in this article, or claim that may be made by its manufacturer, is not guaranteed or endorsed by the publisher.
